# Recent Advances in Wearable Textile-Based Triboelectric Nanogenerators

**DOI:** 10.3390/nano14181500

**Published:** 2024-09-15

**Authors:** Sivasubramaniyan Neelakandan, S. R. Srither, N. R. Dhineshbabu, Suman Maloji, Oscar Dahlsten, Ramachandran Balaji, Ragini Singh

**Affiliations:** 1 Department of Chemistry, Sri Sai Ram Institute of Technology, West Tambaram, Chennai 600044, Tamil Nadu, India; 2 Centre of Excellence for Nanotechnology, Department of Electronics and Communication Engineering, Koneru Lakshmaiah Education Foundation, Vaddeswaram 522302, Andhra Pradesh, India; 3 Department of Electronics and Communication Engineering, T. John Institute of Technology, Bengaluru 560083, Karnataka, India; 4 Department of Manufacturing, Saveetha School of Engineering, Chennai 602105, Tamil Nadu, India; 5 Department of Physics, City University of Hong Kong, Tat Chee Avenue, Kowloon, Hong Kong SAR, China; 6 Department of Biotechnology, Koneru Lakshmaiah Education Foundation, Vaddeswaram 522302, Andhra Pradesh, India

**Keywords:** triboelectric nanogenerators, smart textiles, nanofibers, wearable electronics, human motion energy harvesting

## Abstract

We review recent results on textile triboelectric nanogenerators (T-TENGs), which function both as harvesters of mechanical energy and self-powered motion sensors. T-TENGs can be flexible, breathable, and lightweight. With a combination of traditional and novel manufacturing methods, including nanofibers, T-TENGs can deliver promising power output. We review the evolution of T-TENG device structures based on various textile material configurations and fabrication methods, along with demonstrations of self-powered systems. We also provide a detailed analysis of different textile materials and approaches used to enhance output. Additionally, we discuss integration capabilities with supercapacitors and potential applications across various fields such as health monitoring, human activity monitoring, human–machine interaction applications, etc. This review concludes by addressing the challenges and key research questions that remain for developing viable T-TENG technology.

## 1. Introduction

When rubbing certain materials together, charge separation occurs. With records dating back to Plato 360 BC, this triboelectric effect is likely to be the earliest way in which mankind encountered separated charges. The triboelectric effect is also important and may even have played a fundamental role in creating life through amino acid generation by spark-activated chemical reactions in charged volcanic plumes [1,2]. As evidenced by the seminal work of Wilcke, van de Graaff, and others, it is possible to exploit this effect to create high voltage-low current triboelectric generators [3,4]. With recent advances, new possibilities and uses are emerging. Owing to significant developments in electronic textiles (e-textiles), electronic elements can now be integrated into textiles in the form of fibers, yarns, and/or textile materials, through economical and well-established routes. In recent decades, rapid developments in the market for wearable and portable devices have spurred micro- and nano-science research to address the energy supply problems of these systems.

The human body is a natural alternative power source for wearable electronics. Footsteps or arm movements, for example, generate enough kinetic power to in principle drive many portable devices [5]. As textiles are used in a variety of areas, including indoor use, transport, engineering, and medicine, practical solutions for harvesting energy from textiles are highly sought. Several studies into wearable photovoltaic textiles, thermoelectric textiles, and wearable piezoelectric generators have been reported [6,7,8,9,10,11,12,13]. However, there are significant problems in matching the technology with real applications. For instance, piezoelectric generators depend on stress, whereas textiles are conformal materials such that the load-transfer strain is minimal, resulting in low power outputs.

In 2012, Z.L. Wang’s research group accordingly suggested new approaches to using triboelectricity for converting mechanical energy into electricity. A flexible triboelectric nanogenerator (TENG) was demonstrated [14]. Extensive investigations into the performance and applications of TENGs followed. It has been shown that TENGs can produce power from large-scale ambient motion by harnessing energy from winds [15,16,17], water [18,19], or from small-scale vibrations, such as human movements [20,21,22] and sounds [23,24]. This method is ideal for textiles, as instead of connecting stress to an active material such as a piezoelectric power generator, it harnesses sliding motion, compression, or stretching. Zhong et al. introduced truly textile-based TENGs (T-TENGs) in 2014 [25]. They consisted of two twisted cotton yarns that could transform biomechanical movements/vibration energy into power and electricity, e.g., for mobile health-monitoring systems. T-TENGs continue to develop and are proving promising for smart textiles, owing to their flexibility, multiple possible motion harvesting modes, good air permeability, and large-scale production capability [26,27,28,29,30,31]. This article therefore reviews recent progress on research into T-TENGs. Although several review articles report the use of flexible TENGs [32,33,34], this review article specifically deals with different aspects of textile-based TENGs. The first is to discuss the way in which T-TENGs operate and the underlying principles. Secondly, textile (fabric and fiber) production methods are described; these have a crucial impact on the choice of T-TENG materials, higher-level assembly processes, as well as cost-effectiveness and the associated ability to compete with battery-technology. Recent T-TENGs are categorized according to manufacturing methods, and their resulting power-densities tabulated and compared. Possible technological applications are discussed.

## 2. Different Modes of Operation of T-TENGs

TENGs convert mechanical energy into electricity using a combination of triboelectrification and electrostatic induction. After frictional contact between two distinct materials, the material with a positive electron affinity is most likely to be a donor of electrons, and the material with a negative electron affinity is most likely to be a receiver of electrons [14]. A force, here in the form of a human movement, can separate the materials and thus create a potential difference between electrodes that are attached to the surface of the materials. The free electrons in the electrodes then flow into the external circuit to balance the induced potential difference and maintain the electrostatic equilibrium. 

**Figure 1:**
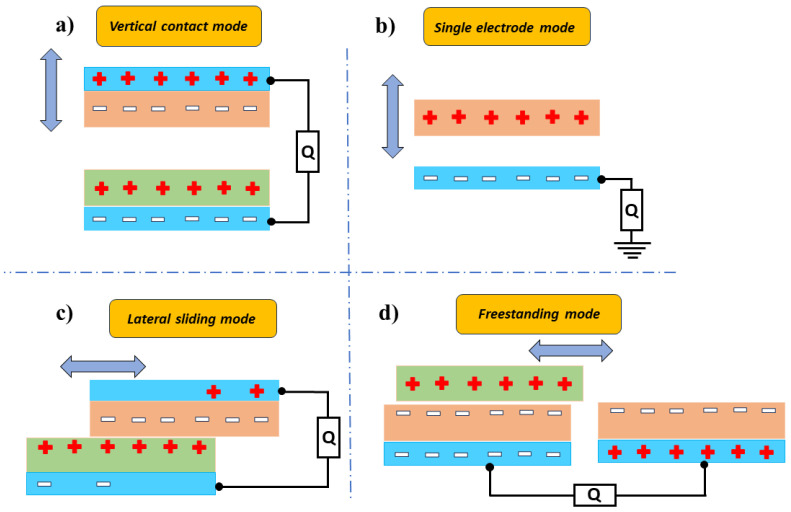
Four basic operation modes of TENGs. (**a**) Contact Separation. (**b**) Single Electrode. (**c**) Lateral Sliding. (**d**) Freestanding Triboelectric mode. Loads are depicted as resistors, connected to electrodes (or ground). The electrodes are coated on tribo-positive and tribo-negative materials, which move relative to each other.

A TENG has at least four modes of operation with different device configurations and numbers of electrodes. These are shown in Figure 1. We now discuss each mode in turn and how they relate to T-TENGs. 

### 2.1. Contact Separation (CS) Mode 

Figure 1a shows the contact separation mode, which consists of two separate tribo-layers facing each other. In this mode, the electrode is coated on the top and bottom sides of the friction surfaces. The pairing of tribal-positive and tribe-negative material can be both dielectric–dielectric and dielectric–metallic. When the external force is acting, oppositely charged surfaces are formed, and when the force is removed, a potential drop is created. When the external load is connected to the electrodes, the excess electrons in the top electrode move toward the bottom electrode, creating the opposite potential and balancing the electrostatic field. When the gap is reduced, triboelectric charge is generated. Therefore, the potential difference disappears and the excess electrons flow back. Most of the examples of this CS mode T-TENGs are based on natural human movements, e.g., they can be built inside shoe soles for human walking [35].

In addition, it can also be embedded inside the fabrics to generate power by pressing or stretching the fabric. The device structure of this type of T-TENGs has been designed in a variety of forms, such as multi-layered fabrics [36,37,38] or yarns [25,39,40]. The output characteristics of the contact separation model can be modeled using a theoretical V–Q−x relationship [41,42] that is given as

(1) $$V(t)=-\frac{Q}{\varepsilon_{o}}\;\left\{\frac{d_{1}}{\varepsilon_{r1}}+\frac{d_{2}}{\varepsilon_{r2}}+x(t)\right\}+\frac{\sigma \;x(t)}{\varepsilon_{o}}$$

where *V* is the voltage between the electrodes, *Q* is the amount of the charge transferred between the electrodes, *x*(*t*) is the separation distance between the dielectric or triboelectric layers at time *t*, σ is the triboelectric charge density, *d*_1_ and *d*_2_ are the thickness of the two respective layers, *ε_r_*_1_ and *ε_r_*_2_ are the respective dielectric constants of the two layers, and *ε*_0_ is the permittivity of free space. 

### 2.2. Single Electrode (SE) Mode

Unlike the other modes of operation, the SE mode has one dielectric material and one electrode connected to the ground (Figure 1b). For practical applications, an additional electrode may be connected to the tribo-material to provide additional mobile electrons without being connected to the lower electrode or the load. Repeated contact and separation between the lower electrode and the tribo-layer causes electrons to move from the main electrode to the tribo-layer (and to any additional electrode attached to the tribo-layer). As the electrical load is attached to only one electrode, the output performance is lower than that of the other modes. However, the single electrode mode has the significant advantage of a reduced need for contact wires, allowing for the tribo-positive material to be unattached to the electrode. Additionally, this mode provides a feature that we can use conventional fabrics or human skin instead of a dielectric material [27,43,44]. 

**Figure 2:**
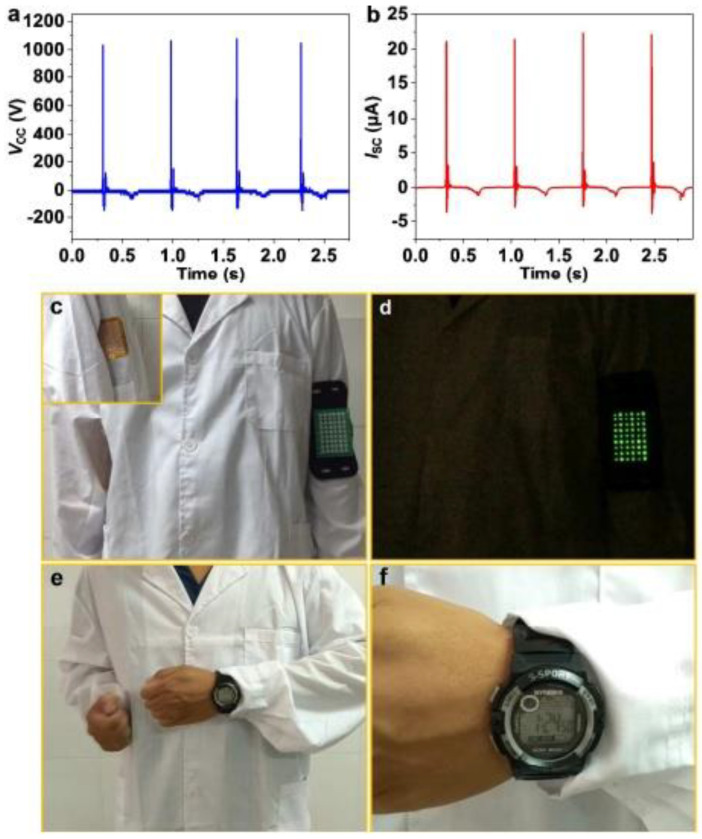
Demonstration of TS-TENG sewn on clothes for mechanical energy harvesting. (**a**) The output voltage and (**b**) current of TS-TENG device (10 cm × 12 cm). (**c**) TS-TENG was connected to a wearable night-time running light. (**d**) Night running light was illuminated by the TS-TENG as the person swinging his arm. (**e**) Digital watch was connected to the TS-TENG without batteries. (**f**) TS-TENG powered a digital watch [45]. Reproduced with permission from [45] (Copyright 2018, Royal Society of Chemistry).

As an example, Ning et al. fabricated washable textile structured single electrode TENG (TS-TENG) using a nanofibrous PTFE film sewn on a cotton lab coat [45]. The nanofibrous PTFE polymer film was initially prepared using a biaxial stretching method and was fabricated using Cu foil to form a single electrode T-TENG. The device with an area of 10 cm × 12 cm was sewn onto the waist of the lab coat shown in Figure 2c. When swinging arms, the device structure delivered peak V_oc_ and I_sc_ at 1050 V and 22 μA, respectively (Figure 2a,b). The study found that the generated power was sufficient to drive a variety of applications, including digital watches and wearable night time running light, shown in Figure 2d–f, respectively. 

### 2.3. Lateral Sliding (LS) Mode 

The construction of a TENG exploiting lateral sliding is the same as that for the case of contact separation; the electrodes are coated at the back of the triboelectric layers. The LS mode tends to create more friction and therefore produces higher power densities than the contact separation mode. The charge generation begins with the force acting in a horizontal direction, as shown in Figure 1c. When the surface of the two materials is in sliding contact, there is a potential difference and the electrons flow from the top electrode to the bottom electrode. If the sliding contact is stopped, the electrons flow back to balance the electrostatic potential. The sliding mode can also be operated rotationally with cylindrical grating structures or disk rotation depending on the applications [46,47]. T-TENGs of this mode are usually constructed with the formation of a grating structure consisting of alternating strips of two different materials, which are attached to the cotton substrate to form the T-TENG.

**Figure 3:**
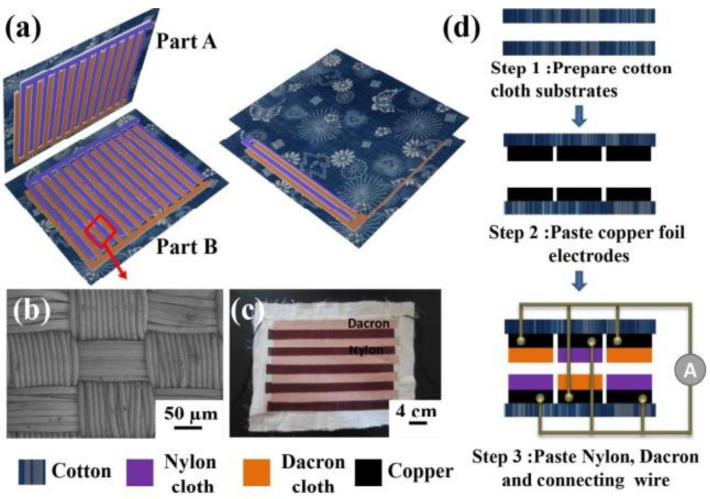
(**a**) Schematic image of a cloth-based TENG device with a grating structure. (**b**) SEM image of the surface of nylon cloth microstructure. (**c**) photographic image of the grid structure of cloth TENG. (**d**) Fabrication process of the T-TENG. Reproduced with permission from [48] (Copyright 2015, American Chemical Society).

Cui et al. have developed a similar type of device structure with real wearable cloths, consisting of grating-structured nylon and Dacron cloth, as seen in Figure 3a [48]. The microstructure surface characterization is shown in Figure 3b. The photograph in Figure 3c displays the grating structure of nylon and Dacron cloth materials. On top of that, the cotton substrate is arranged to form the overall T-TENG as shown in Figure 3d. Alternatively, the nylon and Dacron strips are attached to the top of the cotton fabric substrate where the Cu electrode strip is attached to the cotton cloth. The relative sliding between the two cotton substrates, e.g., when attached to the inner forearm and the waist, causes electrons to flow between the back electrodes of nylon and Dacron materials. With this arrangement, the output values of 2 kV and 0.2 mA were achieved.

### 2.4. Free-Standing Triboelectric-Layer (FT) Mode 

This model consists of a moving object and a pair of patterned electrodes below the triboelectric material, shown in Figure 1d, where the size of the pattern of the electrode is the same as that of the moving object. After triboelectrification between the triboelectric layer and the moving object, as the loaded moving object approaches or moves away, an asymmetric charge distribution is created on each electrode, resulting in electron flow through the external circuit connecting the two electrodes to be neutralized. This mode is also popular in T-TENGs because, similarly to the single-electrode mode, the triboelectric material can move freely without an electrode or electrical connection. The existence of two electrodes in this mode can be associated with better power performance than the single-electron mode. Many of the T-TENGs employing this mode are operated in combination with the contact separation (CS) mode, an approach known as the freestanding-tribo-layer CS mode (FTCS) [27,49]. 

**Figure 4:**
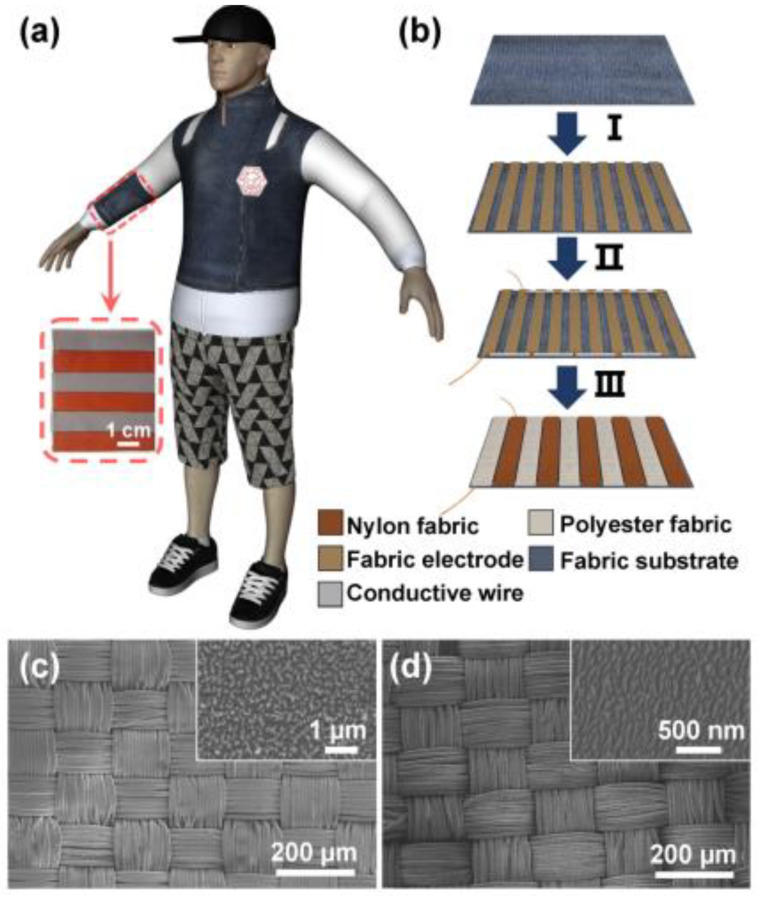
Structure design of fully cloth-based T-TENGs harvesting mechanical energy from human movements (**a**) schematic representative of T-TENG sewn on cloth, inset: photographic image of wearable T-TENG. (**b**) Schematic illustration of the fabrication process. (**c**,**d**) are the SEM images of nylon and polyester fabrics; the insets in (**c**,**d**) are the corresponding partial enlargements of the respective nylon and polyester fabrics. Reproduced with permission from [50] (Copyright 2020, American Chemical Society).

The device structure consists of three triboelectric materials, the first two of which are arranged in strips attached to the electrode on the back or in the core. Here, strips can be a fabric that is woven together to form a cloth substrate. The remaining third material acts as a freestanding layer and typically has an electron affinity between the first two materials, which moves in a vertical direction in contact with and separates from the textile substrate. Based on contact electrification, positive and negative charges formed on the surface of the first two materials. The periodic movement of the third material results in a potential difference between the electrodes, thus generating electrical power. This type of model has been proposed by Zhang et al., [50] who used a fully cloth-based, wearable T-TENG sewn on cloth for human movements, as shown in Figure 4a. The fabrication process is shown schematically in Figure 4b, the nylon and polyester fabric strips were used as two building blocks for the wearable T-TENG. The fabric-based double-sided conductive tape was attached to the cotton substrate, with nylon and polyester strips adhering to the opposite side. The corresponding SEM image confirmed the nanowire pattern for nylon and polyester fabrics, with the partial enlargement insets showing 100 and 50 nm, respectively, shown in Figure 4c,d. In this example, spandex fiber was used as a freestanding layer, V_oc_ and I_sc_ were 170 V and 7.9 µA, respectively. In addition, other types of fabrics such as nylon, Dacron, cotton, and linen have been used to replace spandex fabrics for which the ability to obtain electrons is in the order of nylon > linen > cotton > Dacron [50]. As reported by the author, the output trend is well correlated to the triboelectric series [51].

## 3. Fabrication Methods of T-TENGs

**Figure 5:**
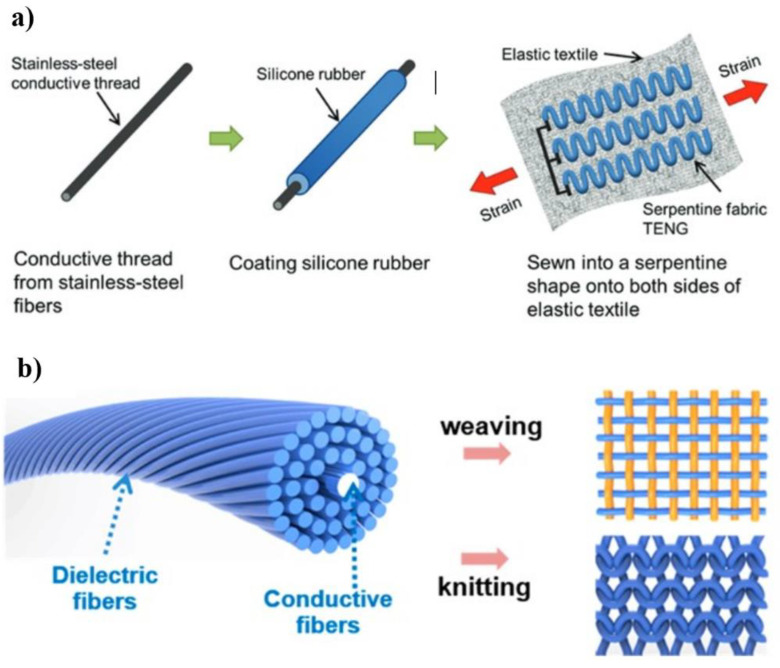
SE mode of fiber/yarn-TENG. (**a**) Fiber-TENG based on silicone-coated stainless steel yarns, reproduced with permission from [58] (Copyright 2017, Wiley); (**b**) core–shell-yarn-based triboelectric nanogenerator by spinning PU fiber with stainless steel fibers, reproduced with permission from [59] (Copyright 2017, American Chemical Society).

Textile production is a huge industrial process, starting with the conversion of natural or synthetic fibers into yarn and finally into fabric. These fabrics are then dyed, printed, and made into cloths after spinning, weaving, knitting, and embroidering for human clothing [52]. E-textiles can therefore also be produced based on carriers such as fibers, yarns, fabrics, and textiles following the same process (Figure 5). There are many e-textile devices confirming the feasibility of using the shapes of fibers, yarns and textiles [53,54,55].

In addition to considering the efficiency and practicality of power generation, T-TENG would only be used in the form of fibers, yarns, and fabrics because it provided better wearability and improved clothing fittings. As regards the design, structure and pattern diversity of triboelectric textiles, TENGs with fibers and yarns are the building blocks for textile devices in which each fiber or yarn is designed to be a single coaxial or twisted 1D TENG. Although it is difficult to deliver high triboelectric outputs when using this single-fiber/yarn device, 1D TENG can be further fabricated into textile TENGs using a knitting/weaving process for self-powered textile applications with higher electrical outputs. In the meantime, some challenges need to be considered while weaving the fiber/yarn TENGs, such as high tensile strength, better wear resistance, and water resistance for the length of the devices. The weaving of 1D TENG for the fabrication of T-TENG can indeed be achieved as a functional finishing on textiles. In contrast, 2D TENGs can be made of fabrics/textiles as single- or multi-layer structures, while conductive or nonconductive yarns or textiles are used as electrodes and triboelectric materials. Two-dimensional structured devices with variable geometric sizes can be achieved and are also promising for large-scale production. Three-dimensional TENG can also be produced by knitting/weaving, braiding, and stitching methods. In most cases, the 3D fabric is made up of 2D fabric, arranged in a stack on top of each other and combined in thickness direction using techniques such as stitching, needle-pinching, chemical bonding, or lamination. However, recent reported T-TENGs are incorporated with polymers in order to achieve triboelectric properties and mechanical endurance, and it is well known that polymers would occupy the porous structure of textiles, which leads to poor breathability and is also not good for the comfort of use. As all we knew that the key elements of the TENG were based on frictional contact between two dissimilar materials with different properties, our aim of the T-TENG design was to bring the two contact surfaces into the textile in a proper manner. Textile production techniques and their structure can have a significant impact on T-TENG performance. To date, several device structures have been invented and can be divided into two groups: T-TENG based on fiber and T-TENG based on fabric. There are also some new designs with special fabric structures, such as 3D-designed TENG. We will introduce these one by one in turn.

### 3.1. Fiber-Based TENGs

**Figure 6:**
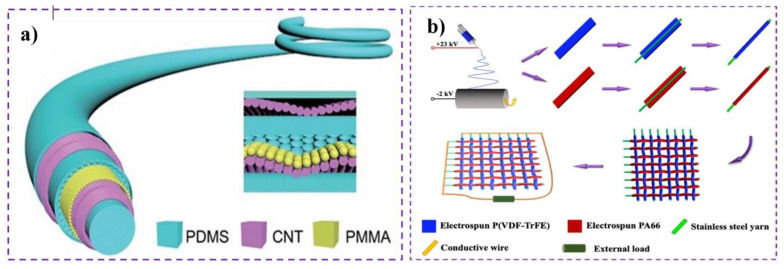
CS mode of fiber/yarn-TENG: (**a**) fiber-TENG prepared by the coating of CNT/PMMA/PDMS on silicone rubber tube, reproduced with permission from [60] (Copyright 2017, Royal Society of Chemistry); (**b**) the fabrication process of the woven-structured TENG, reproduced with permission from [61] (Copyright 2021, Elsevier).

This type of structured TENG usually consists of a single or multiple fiber in which the electrode and the triboelectric material are placed inside or outside the fiber to obtain the relative motion required for the T-TENG. So far, the coaxial structure or core–shell configuration is the commonly adapted design structure for all kinds of fiber-based TENGs, it can be constructed using insulating polymer fibers or conductive wires with a larger diameter for the operation of the device [56]. Usually, the nonconductive polymer fibers serve as triboelectric materials or as an encapsulation layer for the device, whereas the conductive wires or fibers would be used as electrodes. The single-fiber TENG can be operated in SE mode and CS mode. In SE mode, single-fiber TENG consists of a conductive core as an inner layer encapsulated with a dielectric material as an outer shell (Figure 5a,b) [57,58]. In CS mode, single-fiber TENG was designed as a core–shell structure, maintaining a gap between the inner core column and the outer shell tube (Figure 6a,b).

### 3.2. Fabric-Based T-TENG 

In this category, the tribo-layer is made from fabric, often from conventional materials, rather than being tailor-made at the fiber level for the T-TENG purpose. Fabric can be used as a tribo-layer in both the CS, SE, LS, FT, and FTCS modes described above. 

Zhang et al. have fabricated a wearable T-TENG with microrod arrays operating in traditional contact separation mode [62]. Nylon fabric and polydimethylsiloxane (PDMS) were used as a material in which a nanowire array was formed on the surface of the nylon fabric using a reactive ion etching technique, and a lithographic process was used to make the oblique PDMS microarray. The wearable TENG was constructed from nylon fabric, a piece of spandex fiber surfaced on the PDMS film, and a carbon paste painted on the back of the two fabrics to serve as electrodes. When driven by regular mechanical force, the microrod array enabled an open-circuit peak voltage (V_oc_) of 1014.2 V for an area of 16 cm^2^ and a short-circuit current density (I_sc_) of 3.24 μA/cm^2^ with a maximum peak power density of 211.7 μW/cm^2^. For practical applications, the wearable T-TENG was first sewed onto a piece of spandex fabric and then attached to different parts of the body—such as knee joints, shoulders, and elbows—to effectively harvest body motion energy.

Several woven T-TENGs have recently been realized. In Reference [63], nylon and Teflon fabrics were used in the form of a textured multifilament. Various samples of woven fabrics such as 1/1 plain weave, 2/2 matt weave, 3/1 twill, and 5/1 twill were first woven and then finally stitched with Ni fabric to form the T-TENG (the notation V/H denotes how many threads are included in between each crossover, vertically V and horizontally H). It was found that the maximum voltage output of a 1/1 single weave of 2.52 V is increased to 10.93 V if using a 5/1 twill weave structure due to greater cumulative contact area owing to fewer crossovers. 

**Figure 7:**
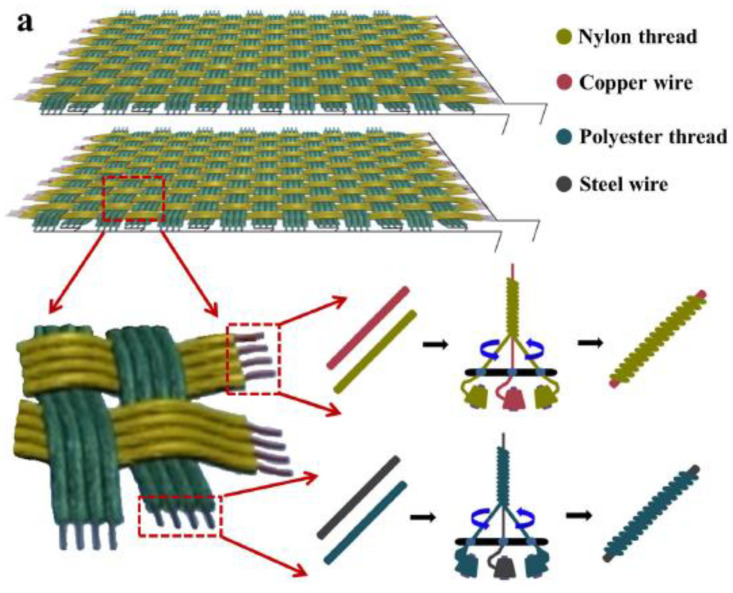

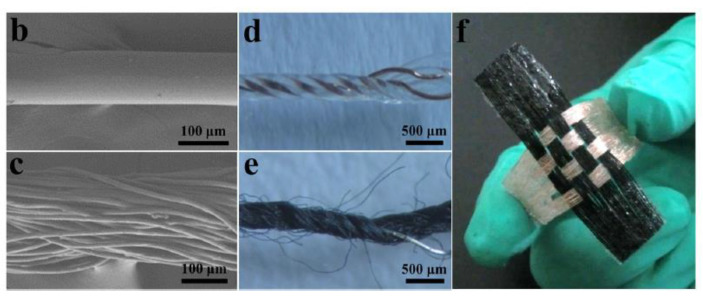
Fabrication of the 2DW-WTNG core shell structure. (**a**) The schematic diagram of the weaving process. Scanning electron microscope images of nylon (**b**) and polyester (**c**) fabrics, respectively. (**d**,**e**) Optical image of nylon coated copper wire and polyester coated steel wire. (**f**) 2DW-WTNG optical image. Reproduced with permission from [64] (Copyright 2019, Springer Nature).

**Figure 8:**
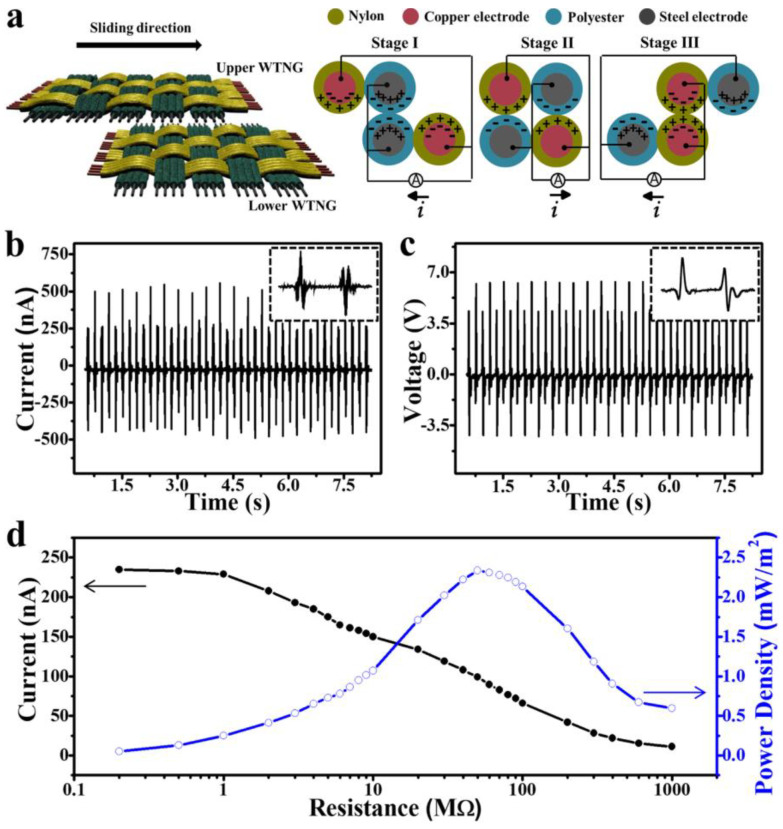
Power generation characteristics of 2DW-WTNG. (**a**) Illustrating the process of generating electricity. (**b**,**c**) 2DW-WTNG output voltage and current. (**d**) Power density at different load resistances [64]. Reproduced with permission from [64] (Copyright 2019, Springer Nature).

Another type of 2D woven wearable T-TENG (2DW-WTNG) was reported by Liu et al. [64]. In the majority of cases, for this type of structure, the construction of the fibers or strips was first modified with conductive fibers and then woven into the final form. Similarly, for their work, nylon thread and polyester thread were stitched with enameled copper wire and steel wire to produce nylon/copper composite conductive fiber and polyester/steel composite conductive fiber. Figure 7a shows the weaving process of 2DW-WTNG. Figure 7b–f shows scanning electron microscopic images and optical images of nylon and polyester fabrics. The nylon and polyester threads were folded and wrapped around the center of the encapsulated copper wire and steel wire for the core–shell structure. Its mode of operation is relative sliding, as shown in Figure 8a. The power generation characteristics of 2DW-WTNG are shown in Figure 8b,d. When force was applied to generate back-and-forth movement (at a sliding speed of 0.15 m/s), a maximum of 6.3 V was obtained at the output current of 575 nA for the applied frequency of 2.7 Hz. A maximum peak power output of 2.33 mW/m^2^ was achieved at a load resistance of 50 M. Powering of LEDs and low-power products was achieved with the use of an intermediate capacitor building up energy.

**Figure 9:**
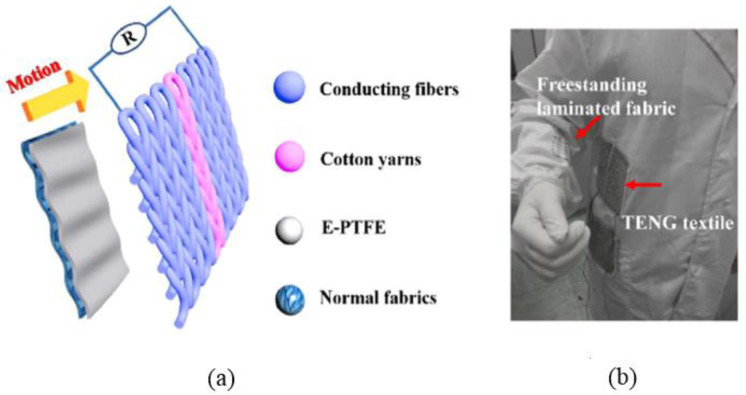
(**a**) Schematic structure of laminated fabrics and TENG textiles. (**b**) Optical image of TENG textile and freestanding laminate fabric is sewn on cloth for energy harvesting from human motion. Reproduced with permission from [65] (Copyright 2019, Elsevier).

Huang et al. [65] reported a T-TENG fabricated with conventional cloth (polyester fabric) knitted from Ag-plated nylon threads, which could serve as both a positive triboelectric material and a charge collector. The laminated fabrics were made from PTFE bonded to nylon fabrics. When the conventional textile was combined with the laminated fabrics, free-standing triboelectric T-TENGs were realized (see Figure 9). The resulting T-TENG voltage and current values were 800 V and 15 μA, respectively, when the pressure was 5 MPa. They also demonstrated a wide range of applications, such as powering warning lighting, a digital watch, and performing human motion tracking and recognition based on harvesting energy from different types of human motion. The reference claims that the materials used are washable and that the manufacturing method is suitable for scaling up to an industrial production line.

In another study, polypropylene non-woven fabrics (PP-NWFs) were prepared and combined with commercial polyamide 66 fabrics to produce T-TENG on a large scale [66]. In that study, polypropylene particles were initially dried at 100 °C for 24 h and then melted using a melt-blowing machine to obtain a rolled PP-NWF. The conductive Ni fabric was then coated with each of the tribo-layers to serve as an electrode. To obtain a larger area, different dimensions of T-TENG were tested. It was found that increasing the size of T-TENG from 30 × 30 mm^2^ to 100 ×100 mm^2^, V_OC_, I_SC_, and Q_SC_ could be significantly increased by approximately 4, 4.7, and 5.4 times from 101.6 V, 9.3 μA, and 34.6 nC to 403.7 V, 43.8 μA, and 186.1 nC, respectively. The device powered LEDs, charged capacitors, and powered an electronic watch.

In recent years, many works have been done using fur materials due to their nanofiber structure possessing high tribo properties. Widespread utilization of these pile-based wearable TENG is hindered by high cost, low efficiency, and difficulties in mass manufacturing. Y. Shen et al. proposed and designed a knitted pile structured T-TENG (wool yarn and polyamide) with outstanding properties for intelligent home sensing and different mode energy harvesting. This smart fabric with a high-density 3D pile structure (approximately 16,128 piles per cm^2^) provides a higher contact area between the wool pile fabric and PET pile fabric, of which the instantaneous peak power density can reach up to 1.4 W m^−2^ under the compressing state [67].

### 3.3. T-TENGs with Fiber-Based Structure 

**Figure 10:**
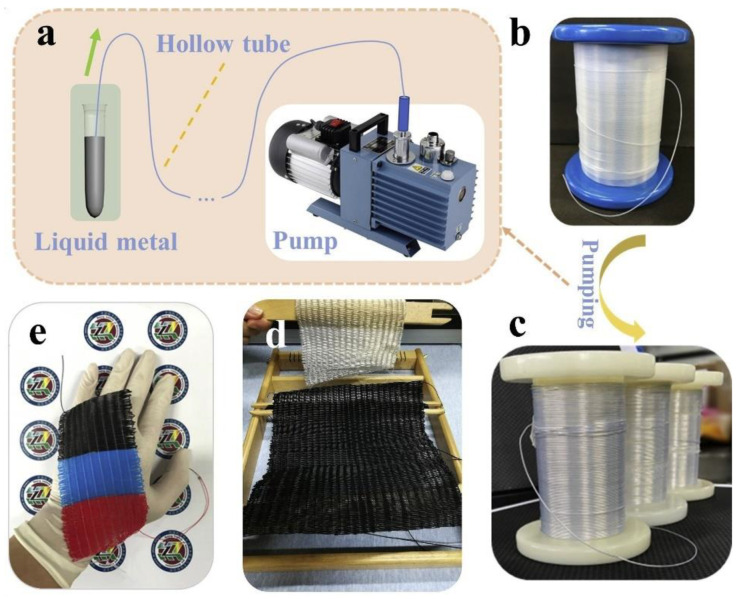
Schematic illustration and fabrication procedure of T-TENG. (**a**) Fabrication process of the liquid metal/polymer core/shell fiber (LCF) structure. (**b**–**e**) Digital photograph (**b**) original ultra-fine hollow fiber polymer; (**c**) hollow fiber polymer after pumping process, (**d**) T-TENG weaving and (**e**) tri-color PTFE fibers. Reproduced with permission [68] (Copyright 2020, Elsevier).

This type of structure usually consists of a single or multiple fiber form in which the electrode and the triboelectric material are placed inside or outside the fiber to obtain the relative motion needed for the T-TENG. Wang et al. proposed a new liquid metal/polymer core/shell fiber (LCF) structure that was achieved by pumping the liquid metal into uniform ultrafine hollow polymer fibers [68]. T-TENG was prepared by simply weaving the fabrics with the resulting LCF structure. The construction steps are shown in Figure 10a–e.

**Figure 11:**
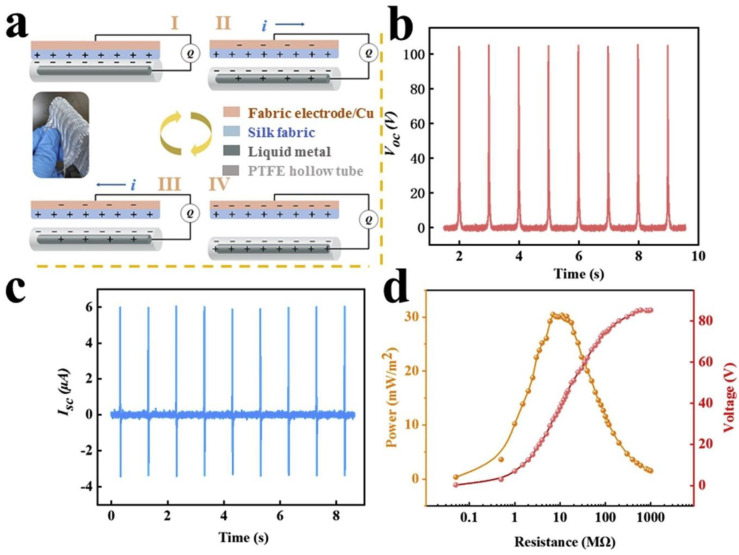
Working principle and electrical output of the T-TENG structure. (**a**) operation of the device in a contact separation mode. (**b**–**d**) electrical outputs, including V_oc_, I_sc_ and power density at different load resistances. Reproduced with permission from [68] (Copyright 2020, Elsevier).

Figure 11a shows the operation of the T-TENG when the silk fabric was stitched onto the fabric electrode. Electrical measurements were made using a linear motor for which the V_oc_ and I_sc_ were 105 V and 6 μA for the applied frequency of 1 Hz. A device with an area of 6 × 8 cm^2^ had a maximum power density of 30.4 mW/m^2^ with an external resistance of 7 MΩ (see Figure 8b–d). As proof of concept, the device was further developed in a single-electrode mode and achieved a peak voltage of 206 V and a peak current of 28.7 μA from tapping with cotton gloves. A smart finger-touch-sensing system was designed for smart home switching control purposes.

There are several reports on friction surface modification to maximize contact area and improve output performance. One study showed that the rough texture of the embroidered surfaces the researchers fabricated showed an improved output performance [69]. In their work, rayon and polyester fiber were used to make a pile-embroidered fiber (roughened texture) as a surface contact for wearable applications. Satin-embroidered fiber (flat textured) was also prepared for comparison against the pile-embroidered surfaces. It was observed that the pile-embroidered surface easily forms a deformable structure when the two textile fibers are in contact and separated, whereas it was not found on the satin-embroidered surface. Under a 9 N compressive force and 1 Hz frequency, the peak output voltage and current density values of the pile-embroidered fibers were 25 V and 0.17 μA, whereas those of the satin-embroidered surface were 6.5 V and 0.04 μA, respectively (8.5 cm × 5.5 cm). This stronger performance is consistent with the pile-embroidered fiber enabling a deformable structure that increases the contact area, helping to maximize output compared to the stain-embroidered fiber that in contrast provides a non-deformable structure.

In T-TENG, the integration of core layer yarn and sheath yarn creates a versatile and robust yarn structure that is pivotal in advanced textile technologies, especially in energy-harvesting applications. This dual-layer system not only enhances the electrical properties necessary for applications like TENGs but also ensures durability and usability in various environmental conditions. Y. Maio et al. investigated how the weaving structure of fabrics can influence the performance of T-TENGs, including both direct-weaving yarn TENGs and those that undergo post-coating. Twelve fabrics with five dissimilar sets of parameters were designed by using a single yarn with a core-sheath structure and PTFE filaments as the sheath yarn. They found that space gapping (1–6 times the parallel space gapping) in T-TENG plays a crucial role in increasing the electrical performance. It improves the efficiency and applicability of T-TENGs in capturing biomechanical energy and monitoring physiological signals [70].

### 3.4. Fabrication of Nanofibers for TENG

We now discuss recent progress in fabrication methods using nanofibers for TENGs (including T-TENGs). A nanofiber (NF) is a fiber with a diameter in the nanometer range. High surface charge density might have been found in electrospun-triboelectric polymer materials with high specific surface area and surface roughness. Here, the fabrication methods using nanofiber for TENGs have been categorized with polymer-based fabrication. They discuss methods that use polymers like PDMS, poly(vinylidene fluoride) [71], polyimide (PI) [72], Nafion, and poly(3,4-ethylenedioxythiophene): polystyrene sulfonate (PEDOT:PSS) and polyvinyl alcohol (PVA), polyamide 6 (PA6), etc. Additionally, nanofillers like nanotubes and nanowires may be aligned inside electrospun fibers using electrospinning processes, which is helpful for TENG-based wearable electronic applications [73].

Recently, the fabrication of 1D nanomaterials of polymer NF, which is also a nonwoven nanotextile, has been undertaken using electrospinning (ES) techniques with diameters ranging from tens of nanometers to several micrometers. The versatile ES method is proving suitable for novel energy applications such as supercapacitors, lithium-ion batteries, dye-sensitized solar cells, photovoltaic cells, fuel cells, and nanogenerators (piezo/tribo) due to the attractive properties of NFs (lightweight, controllable pore structure, high surface-to-volume ratio, and good mechanical properties) [35,74,75,76,77,78,79,80]. We therefore now describe certain recent studies related to NF TENGs.

Zhang et al. [71] created a micrometer length NF of poly(vinylidene fluoride) (PVDF) with heart-like nodules for a wearable T-TENG using ES techniques. A strong performance of the device, consisting of two tribo-materials, was achieved using the contact separation mode. On one side, a PVDF NF mat (tribonegative layer) was deposited on the conducting fabrics and on the other side, a natural rubber mat was deposited on the same conducting fabrics. The charge transfers between the two triboelectric materials following gentle hand patting. The output performance of the device (working area: 20.25 cm^2^) showed high peak voltage (1063 V), peak current (196 µA), and peak power density (14.8 Wm^−2^) and it could power 595 LEDs, a scientific calculator, and a timer. In another study, EN PVDF-co-trifluoroethylene (-TrFE) was deposited on an ITO-PET (40 × 40 mm^2^) substrate with various diameters, such as 310, 260, and 210 µm, and the performance was investigated by Jang et al. using various experiments for TENG [81]. An applied force (~10 N) with frequency (2 Hz) was applied to the contact and separation modes of a triboelectric device. The results of the EN-TENG experiment showed an increase in the open-circuit voltage (from 86.1 to 576.7 V) when optimizing parameters such as working distance, needle gauze and electrospinning time, with a power density of ~2.39 W/m^2^ at a load resistance of 100 MΩ (R_L_), demonstrated by the illumination of 200 LEDs.

**Figure 12:**
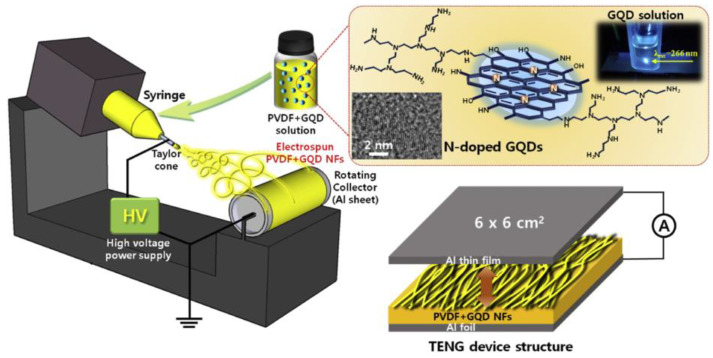
Illustration of the fabrication process of PVDF/GQD composite NFs by the ES method and diagram of the TENG device structure. Structure of N-doped GQDs and their luminescence images under UV excitation (λ ~266 nm). The inset shows high-resolution TEM images of GQDs. Reproduced with permission from [82] (Copyright 2019, Elsevier).

Choi et al. [82] fabricated composite NF using PVDF/graphene quantum dots (GQD) by the ES technique for TENG. The surface-modified nitrogen and GQD at the volume ratio of 0, 2.5, 5, 7.5, 10 vol% were added into PVDF homogeneously to produce the composite NF deposited on aluminum (Al) foil (6 × 6 cm^2^). Thin Al film was used as another electrode for triboelectrification (see Figure 12). The results showed that as the concentration of GQD increased from 0 to 5 vol%, the maximum output power of the composite material TENG device increased from 35 to 97 µW (R_L_ = 20 MΩ). In comparison, the addition of GQD above 5 vol% tends to decrease the output value. As a result, the optimal amount of GQD incorporation into PVDF NF stimulated polar β-phase formation and enhanced TENG performance in this study. 

One-step fabrication of ES polyimide (PI) NF membranes with concentrations between 5 and 20 wt.% with an interval of 5 wt.% for TENG was reported by Kim et al. [72]. For the construction of TENG with contact and separation modes, PI NFs were placed on an ITO-PET substrate (40 × 40 mm^2^) as a bottom layer and Al as a top layer with applied force and frequency of 10 N and 2 Hz, respectively. The output performance of the maximum voltage, current, and power increased with an increase in the PI concentration, such as 753 V, 0.79 µA and 2.61 Wm^−2^ at R_L_ = 100 MΩ, which was demonstrated by powering 55 commercial green LEDs. Kong et al. [83] investigated the churros-like PVDF NFs produced by solvent evaporation kinetics with varying relative humidity (10%, 20%, 45%, 60%, and 70%) using ES techniques for TENG. The flow rate of the solution, working voltage, needle diameter, rotation speed of the collecting drum, and working distance were 2.0 mL/h, 13 kV, 23-gauge metal needle, 200 rpm, and 16 cm, respectively. From the observed result, it was found that the relative humidity increases with the surface area and β-phase ratio of the PVDF NF. Similarly, the output performances of the maximum voltage, current, and power dramatically increased from 14 to 234 V, from 0.5 to 11 μA, and from 5 to 1738 μW cm^−2^, respectively, which was demonstrated by powering an array of 60 series-connected LEDs.

## 4. Integration of T-TENG with Textile Energy Storage Device

**Figure 13:**
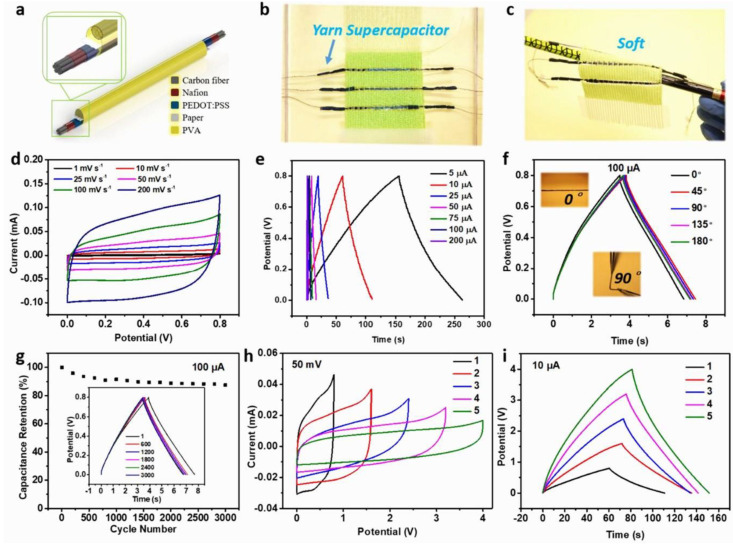
Schematic representation and electrochemical characterization of the yarn SC. (**a**) Schematic view of the structure of the yarn SC. (**b**) The optical image of the fabric SC woven by the PA yarn and the yarn SC. (**c**) An optical image showing the fabric SC hanging on a pencil. (**d**) CV curves of the yarn SC at different scan rates. (**e**) Charge–discharge profiles at different current loads. (**f**) Charge–discharge curve data bending from 0−180° degrees. (**g**) Stability cycles of the yarn SC. (**h**) CV curves in series connection. (**i**) Charge–discharge profiles in series connection. Reproduced with permission from [84] (Copyright 2020, American Chemical Society).

For many energy harvesting applications, one builds up energy on a small storage device and then powers the application in question once there is sufficient energy. As charging a capacitor requires DC, most traditional TENG fabrics use rectifier bridges to convert AC to DC. In one of the studies, a DC fabric-TENG (DC F-TENG) with a planar structure was instead designed to convert the bio-motional energy into DC power by employing the electrostatic breakdown phenomenon of cloths [84]. With a small TENG area of 1.5 cm × 3.5 cm up to 416 LEDs were easily illuminated when connected in series and a 6.8 cm × 7 cm generated peak values of 4500 V, 40 μA, and 4.47 μC per motion cycle. A yarn supercapacitor (SC) was fabricated with a bunch of carbon fibers coated with Nafion and poly(3,4-ethylenedioxythiophene): polystyrene sulfonate (PEDOT:PSS) and polyvinyl alcohol (PVA) gel as shown in Figure 13a. In addition, the proposed flexible yarn SC can be easily woven as a fabric SC or made with DC F-TENG to be used as a self-charging power system. Figure 13a–c show the image of the built yarn SC, which is visibly soft and flexible. Electrochemical studies such as cyclic voltammetry (CV), charge–discharge, and electrochemical impedance spectroscopy were conducted to confirm the capacitive storage performance. As shown in Figure 13d–f, the CV results indicate a good capacitive behavior between the potential window, 0 and 0.8 V. The charge–discharge profile shows good capacity retention for a current load of 100 μA. The Nyquist plots show good electrical contact between the fiber networks and the electrolyte. As a result, Chen et al.’s studies have shown that SCs of yarn and fabric can maintain good mechanical and electrochemical performance for our daily needs. 

In another work, Qi et al. [85] demonstrated a stretchable energy-harvesting TENG with enhanced surface roughness. A balloon-blowing technique was employed to prepare a hierarchically wrinkled Polyamide 6 (PA6) membrane. Initially, the polyacrylamide (PAAm) hydrogel film was attached to the surface of an air-filled balloon and then the PA6 nanofibrous membrane was prepared by ES. In this study, it was observed that the hierarchical wrinkled structure increased the surface roughness of the PA6 membrane and therefore achieved a higher voltage and output current of 270 V and 11 μA with a power density of 25 W/m^2^ when the load resistance was 50 M. The stretchable TENG was integrated with the energy management system combined with the Bluetooth data transmission module, such that digital signal of the motion was displayed on a personal computer or smartphone via the Bluetooth module in real time. 

Bayan et al. [86] developed a textile-based triboelectric nanogenerator (T-TENG) by integrating silver nanoparticles loaded on graphitic carbon nitride (g-C_3_N_4_) nanosheets with carbon cloth fibers. By introducing a nylon layer between the nanosheets and carbon fibers, they achieved charge trapping and interface modification, leading to a significant enhancement in the triboelectric properties of the T-TENG. Additionally, temperature-dependent studies showed that the Ag/CN/nylon-Teflon based bi-layers T-TENG remain stable at higher temperatures and exhibit a high-power conversion efficiency of up to 65 °C. This device is capable of generating an open-circuit voltage of up to 200 V and can charge Teflon material under mechanical agitation, positioning it as a promising power source for wearable electronics.

## 5. Application of T-TENGs

### 5.1. Applications in Self-Powered Pressure Sensors

T-TENGs can be used as self-powered pressure sensors. One of the works by Zhao et al. [87] reported a machine-washable and breathable pressure sensor based on TENG textiles. The copper-coated polyacrylonitrile (Cu–PAN) and parylene–Cu–PAN textile yarns were loaded onto an industrial knitting machine for knitting. A smart glove was used for gesture recognition in the study. A circular pressure sensor was designed with a size of 1 cm^2^ stitched to the gloves positioned at the fingertips of all five fingers, and a large area of 4 cm^2^ was positioned at the palm. The test was shown to hold a pen, an egg, and a smart glove dumbbell. While holding the pen, pressure detected only the part of the thumb and the index finger; no signal was received from the other parts of the smart gloves. Likewise, while holding an egg or a dumbbell, voltage was generated at all the points, with distinguishable differences between the different sensors.

A similar type of application has been reported by Ma et al. [88], who manufactured single-electrode triboelectric yarn capable of harvesting biomechanical energy and sensing multifunctional posture from human motion. The TENG was designed as a textile-sensing pattern for obtaining pressure-sensing information. By touching a finger to the sensing array, the electrical signals of the sensor arrays were monitored by arrays capable of detecting the location of the pressure and the magnitude of the applied load. The study confirmed that the simple yarn could be used to develop a multifunctional, wearable electronic textile that has potential applications in the mass production of smart textiles. 

### 5.2. Other Different Applications 

Fan et al. [89] demonstrated a washable triboelectric textile sensor array for health monitoring. They developed a wireless mobile health monitoring system combined with a triboelectric sensor array to continuously acquire physiological signals. The fabricated sensor array was stitched into multiple parts of cloths to monitor pulse and respiratory signals in real-time. The study provided a high sensitivity to continuous long-term health surveillance with noninvasive assessment for the analysis of cardiovascular disease and sleep apnea syndrome.

In another study, the detection of body motion and antibacterial activity was investigated by Gan et al. [90]. Wearable nanofibrous scaffolding (MWCNT/Ag/PLA nanofibrous membrane) was produced and the membrane detected the sensor signals that were attached to different parts of the body, such as the finger, elbow, knee, and forehead. It was found to react quickly, precisely, and consistently to all kinds of body movements. An electrical resistance response of 1.0–1.62 R/R_0_ was observed for 50 folding–unfolding cycles in one of the finger-attached membrane studies, in which R is the electrical resistance and R_0_ is the initial resistance. The material has the added advantage of being antibacterial. The antibacterial study confirmed that the nanofibrous membrane has an antibacterial effect with inhibition zones of 0.48 cm against gram-negative Escherichia coli and 0.45 cm against gram-positive Staphylococcus aureus.

Self-powered sensors based on TENGs have shown great advantages in human-machine interactions. Low triboelectric output and low sensitivity due to miniaturization lead to a reduction in the TENG contact area and thus lower output performance. In addition, the use of a two-electrode system requires a support structure between the two TENG layers to achieve contact and separation, making the structure complicated and less convenient in terms of wearability. To overcome the aforementioned drawbacks, Zhang et al. [91] developed a 3D-printed smart glove with pyramidal MXene/Ecoflex-composite-based toroidal TENG for wearable human–machine interaction applications. Ecoflex is used to create a conductive fabric and is uniformly distributed on MXene. This combination results in the MXene/Ecoflex nanocomposites, which exhibit high negative charge characteristics, excellent external pressure response output, and high sensitivity. The pyramidal MXene/Ecoflex composite-based toroidal TENG not only exhibits a maximum peak-to-peak voltage of 19.91 V but also demonstrates excellent electrical stability and durability, with its electrical output voltage remaining essentially constant with no significant fluctuations for 50,000 consecutive cycles of the contact separation process at a frequency of 5 Hz. The developed sensor successfully captures the movements of the fingers in human–machine interaction applications.

**Figure 14:**
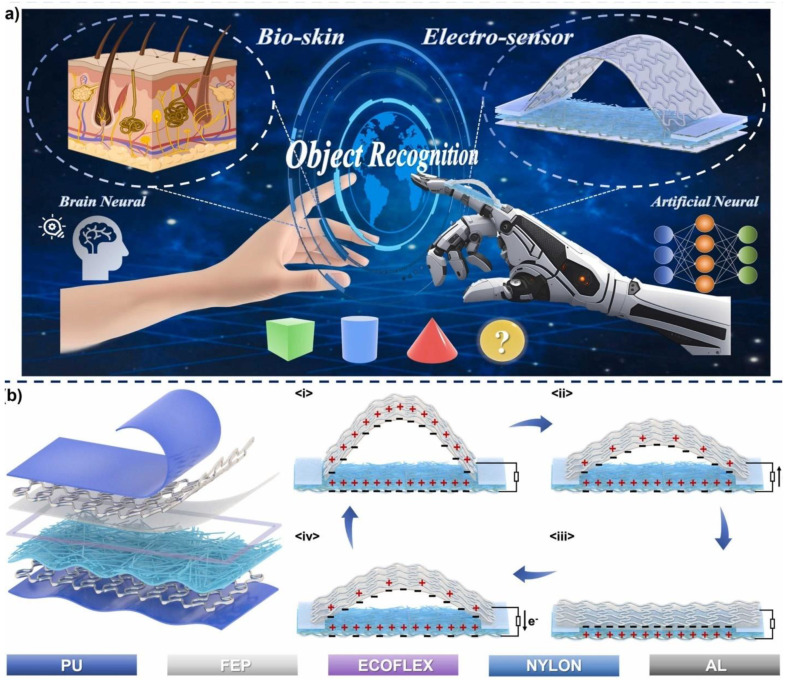
(**a**) The application scenarios of HITWS. (**b**) The structure and working principle of HITWS. Reproduced with permission from [92] (Copyright 2024, Elsevier).

Yang and coworkers [92] developed an intelligent triboelectric wearable sensor (HITWS) made from a mica/nylon composite nanofiber film (Figure 14). This sensor has been created using electrospinning techniques and the incorporation of dielectric particles, which significantly enhance its sensitivity and power density compared to earlier models. The inclusion of mica particles is particularly noteworthy, as it improves the dielectric property and surface charge density of the film, positioning it for effective use in object recognition and deep learning applications. In addition to that, mica also enhances the surface roughness and diameter of the nylon films. HITWS sensor shows improved sensitivity (0.082 kPa^−1^) and an SNR of 51.95 dB, and they achieved a high-power density of 11.82 11.82 W/m^2^. The authors claimed that this sensor is beneficial in the field of object recognition, such as for remote interaction, medical prosthetics, and smart robots.

## 6. Summary and Perspectives

Recent progress on T-TENGs based on fabric and fiber has been briefly discussed in our review, and it has been shown that T-TENGs can harvest energy from human movements to realize self-powered motion sensors and potentially power smart textiles. A significant advantage is that almost all of the textile materials we wear today can be used in T-TENGs due to the universality of the triboelectric effect. The reported T-TENGs are generally flexible, stretchable, wearable, and even machine washable. Several parameters, such as surface roughness creation, structural modification or optimization, and textile weaving/knitting/sewing, can be used to effectively improve output performance. Using electro-spun nanofibers can moreover give significantly higher power outputs according to certain studies. T-TENG prototypes are also shown to power small electronic devices such as LED displays and electronic watches and can be used as self-powered pressure and motion sensors, e.g., for health-monitoring applications.

Despite these developments, some challenges remain to be overcome. There is a mismatch between the power output and that required in general. For example, a single signal from a commercially available state-of-the-art wireless transmitter requires about 0.5 mJ. Tapping with a glove as in Ref. [68] produces nearly enough power for this (peak power of the order of 30.4 mW/m^2^). Most smart textile applications will likely require significantly more power, motivating further research. The comparison chart of the power density values corresponding to the triboelectric materials concerning the sample size of the wearable T-TENGs is presented in Table 1. It suggests that optimism concerning further improvements to the power output is warranted. Moreover, there is an issue concerning the power management system (capacitors, rectifiers, etc.). Currently, lithium-ion batteries and non-flexible super-capacitors are commonly used. This hardware also needs to be textile-compatible, motivating further research into flexible and washable supercapacitors. Moreover, the high variability in time and space of the available voltage and current poses a challenge for efficiently extracting power, motivating us and others to undertake research into novel methods for intelligently tailored adaptive power electronics. 

## Figures and Tables

**Table 1:** Summary of the parameters of fabric- and fiber-based T-TENGs.

Samples/Electrodes	Area (cm^2^)	Peak Power Density (μW/cm^2^)	Input Energy	Year	Ref.
Al, wool, PTFE, Ag	96	140	1 Kpa, 1 Hz	2024	[67]
Electrode: MWCNT, PEDOT:PSS, and carbon	130	448	88.5 Kpa, 30 Hz	2024	[93]
Pu, FEP, ecoflux, nylon, Al	8	1182	10 N, 3 Hz	2024	[92]
PDMS, graphene, polyester fabric, nitrile, Cu	9	30	20 N, 15 Hz	2024	[94]
Cu, PET, EcoFlex, bimetallic electrodes	5	299.2	8 N, 8 Hz	2024	[95]
Conductive yarn, cotton yarn, polyester–cotton blended yarn	17.35	-	25 N, 5 Hz	2023	[70]
Polypyrrole, PTFE, Ag	35	-	4 N, 2.5 Hz	2023	[96]
TiAlC_2_, TPU, Al	72	0.16	3 Hz	2023	[97]
PDMS, PET, Al, Ni	-	1.3	16 N	2022	[98]
PVDF-HFP, AgNWs, Mn- BNT-BT, Al	4	4700	100 N, 4 Hz	2022	[99]
Ti_3_C_2_Tx, PEDOT: PSS, Ecoflex, AgNW	2	4.2	10 N	2022	[100]
Carbon fibers, ZnO, PDMS	-	74.1	600 N	2021	[101]
Carbon, silk, BFO- GFF/PDMS	6	151.42	0.5 Hz	2021	[102]
Porous flexible layer (PFL) @ waterproof flexible conductive fabric (WFCF)Electrode: WFCF	8	631,500	20 N, 15 Hz	2021	[103]
Liquid–metal/polymer core/shell fiber (LCF) Electrode: Cu	48	3.04	1 Hz	2020	[68]
Polyimide nanofibers and Al thin film Electrode: Al and ITO	16	261	10 N, 2 Hz	2020	[72]
PVDF nanofibers and Al thin film Electrode: Al	1	1738	-	2020	[83]
Polyamide 6 (PA6), polyacrylamide ((PAAm) LiCl) and silicon rubber Electrode: Cu	2.25	2500	200 Kpa, 3.5 Hz	2020	[85]
Nylon fabric and PDMS electrode: Carbon paste	16	211.7	-	2019	[62]
Nylon and Teflon fabrics electrode: Ni	25	12.84	-	2019	[63]
Nylon/Copper composite and polyester/steel composite electrode: Cu/steel	2.25	0.233	2.7 Hz	2019	[64]
Polyester fabric, nylon threads, and PTFE Electrode: Ag	45	20.3	5 Mpa	2019	[65]
PVDF-TrFE nanofibers and aluminum electrode: ITO and Cu tape	16	239	-	2019	[81]
Silk and PVDF electrode: Carbon fiber	8	310	Hand tapping, 2 Hz	2018	[104]
Nylon and PTFE electrode: MWCNT, PEDOT:PSS and Carbon fiber	17.35	0.8	-	2017	[38]
Silicone rubber and skin Electrode: SS wire	16	8.5	5 N, 2 Hz	2017	[105]
Cotton fabrics and PDMS electrode: CNT	16	3.75	8 Hz	2017	[106]
Ag and silicone rubber electrode: AgNWs and Ag yarn	36	48	10 N, 3 Hz	2017	[107]
Fabric, Skin and silicone rubber Electrode: SS	146.7	95.3	-	2017	[58]
Nylon and PTFE electrode: Graphene	3	5.33	3 Hz	2016	[108]
Fabrics, carbon fiber, and PVDF Electrode: carbon fiber	30	70	-	2015	[35]
Al, fabrics, and PDMS electrode: Al	6	180	3 N, 1.25 Hz	2015	[109]
Nylon and fluorinated ethylene propylene (FEP), electrode: Ag	16	4.65	5 Hz	2015	[110]
